# Supplementary material for: Three-dimensional atomic scale electron density reconstruction of octahedral tilt epitaxy in functional perovskites
Source: Nat Commun. 2018 Dec 6;9:5220. doi: 10.1038/s41467-018-07665-1 (PMC6283878; doi:10.1038/s41467-018-07665-1)
Supplement: Supplementary file 1 — Supplementary Information [file 41467_2018_7665_MOESM1_ESM.pdf]

Supplementary Material

Three-dimensional Atomic Scale Electron Density Reconstruction of Octahedral Tilt Epitaxy in Functional Perovskites

Yuan, *et al*.

Supplementary Table 1 | Comparison between coherent Bragg rods analysis and other imaging techniques.

	CDI	TEM/STEM	COBRA
Probe beam	X-ray/Electron beam	Electron beam	X-ray
Beam coherency requirement	Fully coherent (>scale of sample)	No requirement	Axial coherence >> film thickness
Sample type	Objects with clear boundaries, e.g. nanoparticles	Specially prepared ultrathin sample	Epitaxial thin film systems
Sample size	Hundreds nm to several microns	Sample thickness <100 nm	Film thickness <20 nm
Sample environment	Mostly non-destructive; in-situ/operando feasible	Destructive; in-situ/operando feasible but challenging	Non-destructive; in-situ/operando routine
Beam stability	Very high stability (long exposure time)	Very high stability	Normal (strong diffraction, shorter exposure time)
Coverage of reciprocal space	Low	Not applicable	High
Best resolution achieved	~5.5 nm in 3D	~40 pm in 2D	~40 pm in 3D
Data processing method	Phase retrieval algorithm	Image processing	Phase retrieval algorithm
Data acquisition time	~ 2 hours	~ 2 min per image	~ 6 hours

Supplementary Note 1: Symmetry based crystal truncation rods measurements

In order to reconstruct electron densities (EDs) in real space using Fourier phase retrieval algorithm, coherent Bragg rods analysis (COBRA) method requires the measurement of crystal truncation rods (CTRs) in the whole reciprocal space. This usually requires a large group of CTRs to be collected. In practice, giving the fact that symmetry equivalent CTRs will be identical to each other, therefore, only symmetry inequivalent CTRs are measured and used to recover the diffraction phase information in the whole reciprocal space. Furthermore, the symmetry of the epitaxial systems is usually dictated by the substrates. In this work, substrates $NdGaO_3(110)_{or}$ (or: orthorhombic) and $DyScO_3(110)_{or}$ of bulk space group of *Pnma*, with termination at their $(110)_{or}$ planes, will only retain one of their mirror symmetry for the epitaxial systems, as shown in Supplementary Figures 1a and b. As for LSAT substrate, *4mm* symmetry is retained for $(001)_{pc}$ (pc: pseudocubic) termination (Supplementary Figure 1c). The presence of above symmetries was also confirmed by experiments discussed in Supplementary Note 2. Accordingly, the CTRs measured experimentally are illustrated in Supplementary Figures 1d-i using dark blue dots in reciprocal lattice, while the CTRs recovered by symmetry are indicated using light blue dots. All the (H, K) values are under 2×2×2 pseudocubic notation.

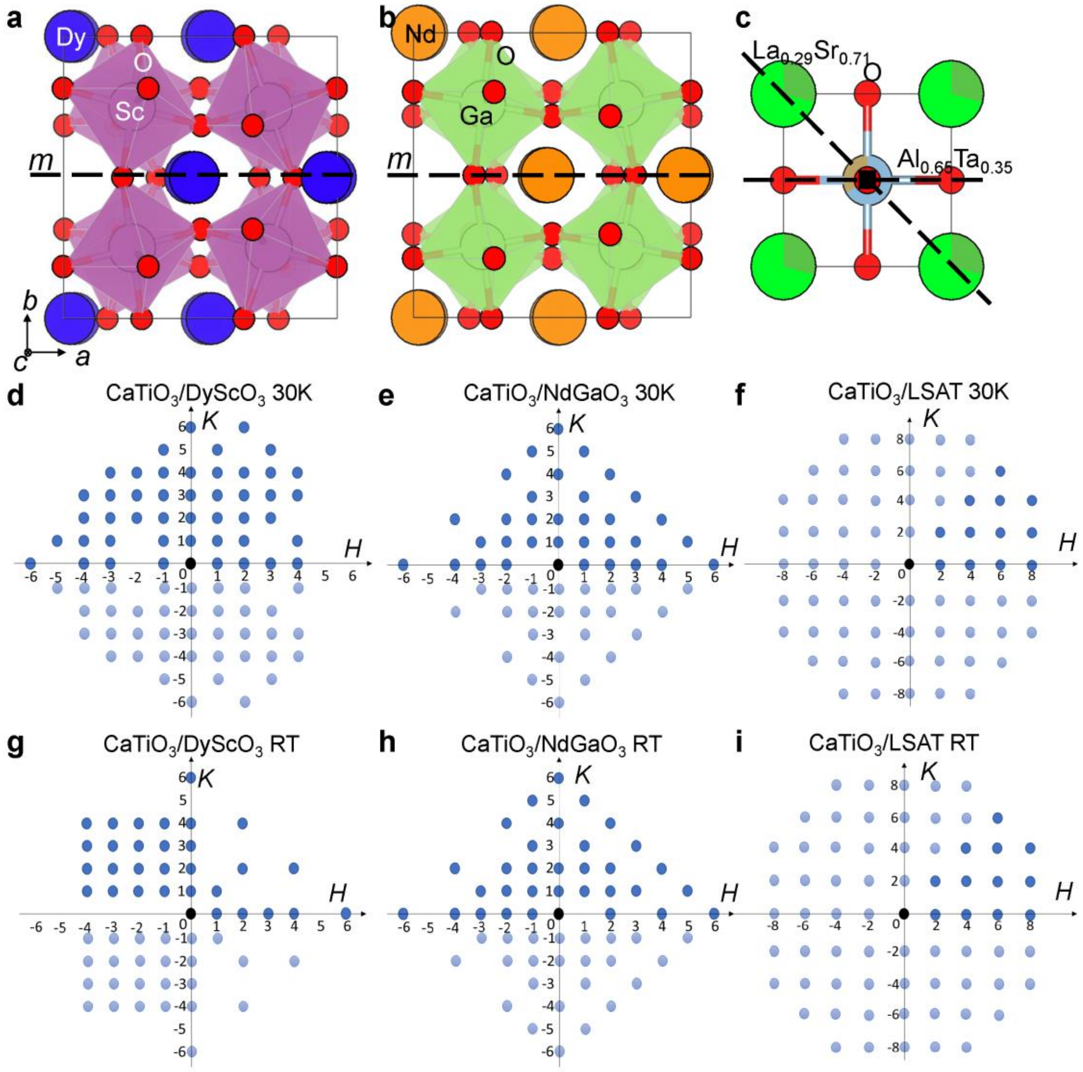

Supplementary Figure 1 | Symmetry of epitaxial systems and experimentally measured crystal truncation rods. Symmetry analysis of terminated substrates show that **a** $DyScO_3(110)_{or}$ and **b** $NdGaO_3(110)_{or}$ has only one mirror symmetry. **c**, $LSAT(001)_{pc}$ has *4mm* point group symmetry. **d-i**, Experimentally measured (*H*, *K*) crystal truncation rods (CTRs) for three different epitaxial systems under 30K and room temperature (RT) are marked by dark blue dots. The symmetry equivalent CTRs in each system are indicated by light blues dots. Experimentally measured (0, 0) specular CTRs are shown using black dots. All the (*H*, *K*) values are under 2×2×2 pseudocubic notation.

Supplementary Note 2: Domain states of epitaxial $CaTiO_3$ on $NdGaO_3$, $DyScO_3$, and LSAT

The domain states of the epitaxial $CaTiO_3$ thin film are analyzed from three perspectives: substrates symmetry and CTRs, optical second harmonic generation (SHG) polarimetry study, and real space structures determined by COBRA and scanning transmission electron microscopy (STEM).

From the substrate symmetry perspective, both $NdGaO_3$ and $DyScO_3$ have the same bulk space group of *Pnma* as $CaTiO_3$. Epitaxially grown $CaTiO_3$ thin films are expected to follow the exact same orientation as substrate to achieve coherent structure across the interface and minimize their interfacial energy. This can be verified by the symmetry of CTRs in these two systems, where only a mirror plane perpendicular to *b* axis exists, giving CTRs equivalence of $(H, K) = (H, -K)$ as shown in Supplementary Figure 2. Using 2×2×2 pseudocubic notation, for $CaTiO_3/DyScO_3$ (Supplementary Figure 2a), (-2,2) rod is equivalent to (-2,-2) rod and inequivalent to (2,2) rod. Similarly, in Supplementary Figure 2b for $CaTiO_3/NdGaO_3$, (2,2) rod is equivalent to (2,-2) rod and inequivalent to (-2,2) rod. As for $CaTiO_3$/LSAT, since substrate LSAT has *4mm* symmetry considering the surface termination, orthorhombic $CaTiO_3$ is expected to form 4 symmetry equivalent domains with in-plane rotation 90º to each other, with similar domain fractions. This will give rise to a macroscopic *4mm* symmetry in CTR measurement, where (2,2), (-2,2), (2,-2), and (-2,-2) rods are equivalent to each other, as shown in Supplementary Figure 2c.

Symmetry of the three epitaxial systems is also verified by the SHG polarimetry study as shown in Fig. 5c and Supplementary Figure 10. A mirror symmetry is observed for $CaTiO_3/DyScO_3$ and

$CaTiO_3/NdGaO_3$, with single domain theoretical fitting to their SHG polarimetry data. For $CaTiO_3$/LSAT, a *4mm* symmetry is observed and polarimetry data is fitted by four equivalent domains with similar domain fractions.

From COBRA reconstructed EDs (Figs. 2a, b) for $CaTiO_3/DyScO_3$ and $CaTiO_3/NdGaO_3$, a coherent oxygen octahedra tilt pattern is observed from substrates to epitaxial thin films, indicating a single domain state of $CaTiO_3$ on these two substrates. This can also be verified by the STEM images shown in Figs. 4a, b, and Supplementary Figure 8. For $CaTiO_3$/LSAT, the successful reconstruction of ED (Fig. 2c) confirms the *4mm* symmetry, which originates from the multidomain nature of the system.

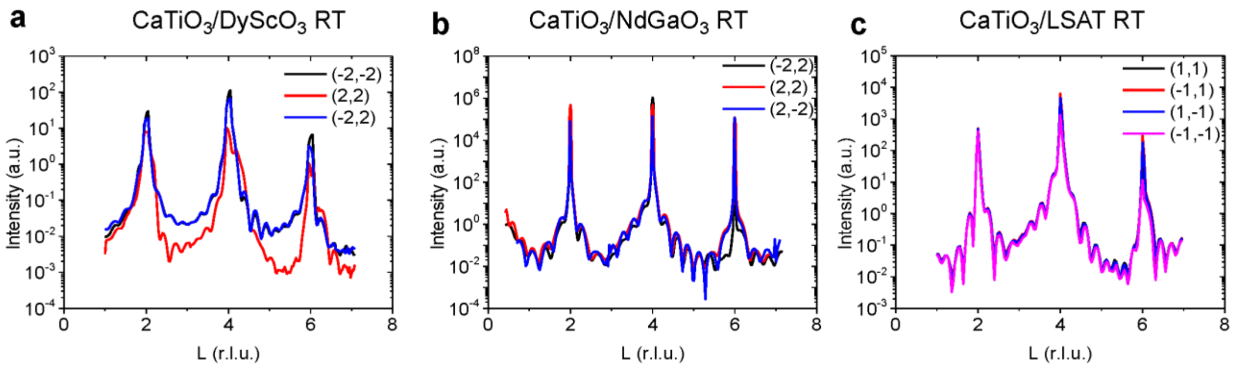

Supplementary Figure 2 | Crystal truncation rods equivalence for the three systems. The symmetry of each system can be identified by the equivalence of crystal truncation rods at room temperature (RT). **a**, $CaTiO_3/DyScO_3$ shows (-2,2) rod is equivalent to (-2,-2) rod and inequivalent to (2,2) rod. **b**, $CaTiO_3/NdGaO_3$ shows (2,2) rod is equivalent to (2,-2) rod and inequivalent to (-2,2) rod. **c**, $CaTiO_3$/LSAT shows (2,2), (-2,2), (2,-2), and (-2,-2) rods are equivalent.

Supplementary Note 3: Folded structure and tilt pattern for $CaTiO_3$ on LSAT

In the cases where thin film and substrate symmetries are different, the epitaxial thin film may form different domains. However, only the CTRs defined by the substrate reciprocal lattice have strong enough diffraction intensities and are measured during experiments. In this case, the reconstructed thin film ED contains thin film structural information that is folded into substrate defined in-plane unit cell. This folding process of $CaTiO_3$ on LSAT is illustrated in Supplementary Figure 3, where four equivalent domains of $CaTiO_3$ are spatially translated by LSAT lattice vectors into one folded structure.

The mathematical representation of this folding process is described as follows. The multidomain ED of thin film ρ^{film} can be written as:

$$\rho^{\text{film}}(\mathbf{r}) = \sum_{i,j} \rho_{i,j}^{\text{film,uc}}(\mathbf{r} - i\mathbf{R}_1 - j\mathbf{R}_2) \tag{1}$$

Where $\rho_{i,j}^{\text{film,uc}}$ is the electron density of the unit cell origins at $i\mathbf{R}_1 + j\mathbf{R}_2$, $\mathbf{R}_1$, $\mathbf{R}_2$ are translational vectors defined by the substrate lattice. The diffraction contributed by thin film can be rewritten as:

$$I^{\text{film}}(\mathbf{q}) \sim \sum_{H,K} | F_{q_1,q_2,q_3}(\overline{\rho_{i,j}^{\text{film,uc}}})\delta(q_1 - HQ_1)\delta(q_2 - KQ_2) | \tag{2}$$

Where F_{q_1,q_2,q_3} is the Fourier transformation (FT) at reciprocal position (q_1, q_2, q_3), Q_1, Q_2 are in-plane reciprocal lattice vectors of substrate, H, K are integers, $\overline{\rho_{i,j}^{\text{film,uc}}}$ is the average ED over i, j. And δ function states the fact that only reciprocal positions along the crystal truncation rods have significant diffraction intensities. Above equations suggest that, the folded ED is the average of

EDs in all unit cells, which are defined by the substrate translational vectors $\mathbf{R}_1$ and $\mathbf{R}_2$, as shown in Supplementary Figure 3.

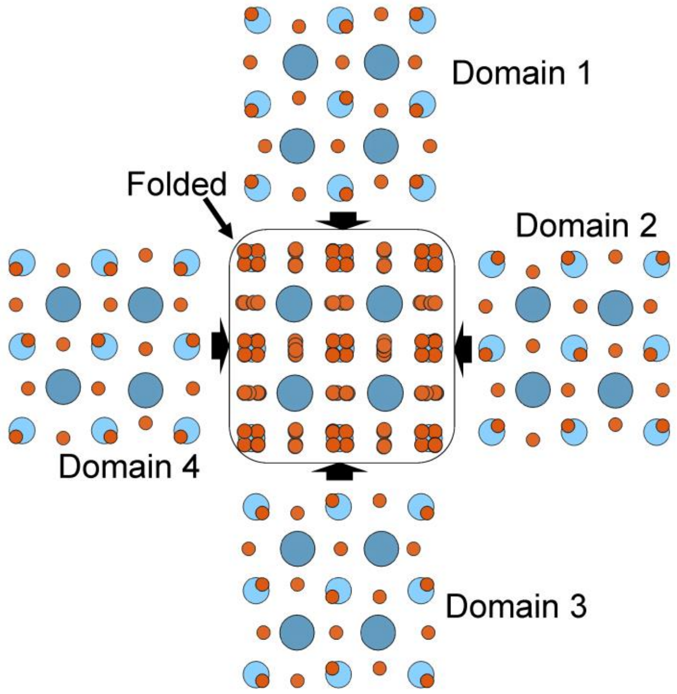

Supplementary Figure 3 | Folded structure of $CaTiO_3$ on LSAT. Four symmetry equivalent domains of $CaTiO_3$ are shown in the figure. The center structure is obtained by spatially translating four domains into single unit cell defined by LSAT in-plane lattice vectors. The Ca, Ti, and O atoms are indicated by dark blue, light blue, and red dots, respectively.

To determine the tilt pattern of $CaTiO_3$ on LSAT, we performed half order peak measurement. According to our measurement, the LSAT substrate has cubic lattice parameters, but it contains small double perovskite structural domains, which give rise to very broad and strong substrate peaks at half order positions (H, K, L = half integers under pseudocubic notation), overwhelming

the weak CTO film peaks associated with all out-of-phase tilts, as seen in Supplementary Figure 4a. Thus, direct determination of out-of-phase tilts is not possible.

However, half order peaks associated with in-phase tilts at (odd/2, even/2, odd/2) or (even/2, odd/2, odd/2) are observed as shown in Supplementary Figures 4c and d, suggesting in-phase tilts about *a*- and *b*-axes. Since CTO film on LSAT has four equivalent in-plane directions and we do not observe a double peak feature, different from the results in ref.[1], these peaks should be explained by multidomain states of a single in-phase tilt along *a*- or *b*-axis (not both).

We also confirm that the absence of (1.5 0.5 4) peak (Supplementary Figure 4b), suggesting an out-of-phase or no tilt along *c*-axis. Hence, we conclude from the half order peak results that the CTO on LSAT only has one in-phase tilt along one of the in-plane directions, and could have out-of-phase tilts or no tilts along the other two axes.

However, COBRA results reveal a finite tilt along the c-axis, ruling out the possibility of c^0. Thus, $a^+b^{-/0}c^-$ or $a^{-/0}b^+c^-$ tilt pattern is expected. Moreover, our density functional theory (DFT) study suggests a tilt pattern of $a^-b^+c^-$ in CTO on LSAT, and the most stable tilt pattern of CTO under a similar strain state (~1%) on NGO substrate is $a^-b^+c^-$. Hence, we conclude that the CTO on LSAT should adopt an $a^-b^+c^-/a^+b^-c^-$ mixed tilt pattern.

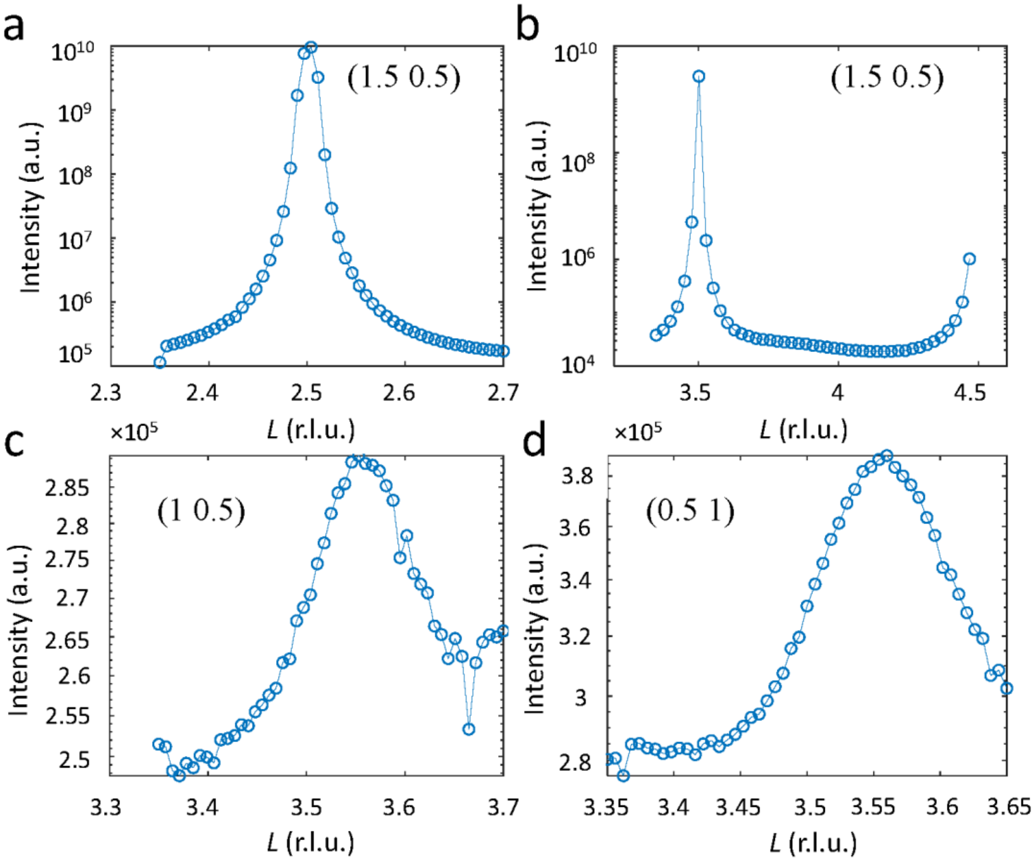

Supplementary Figure 4 | Half order diffraction peaks of $CaTiO_3$ film on LSAT. a Strong (1.5 0.5 2.5) peak from LSAT overwhelm the possible out-of-phase tilt peaks from CTO thin film. **b** The absence of (1.5 0.5 4) peak indicates a c^- or c^0 tilt about *c*-axis. **c-d** The single peak at (1 0.5 3.5) and (0.5 1 3.5) position suggests the CTO on LSAT has only one in-phase tilt along one of the in-plane axes.

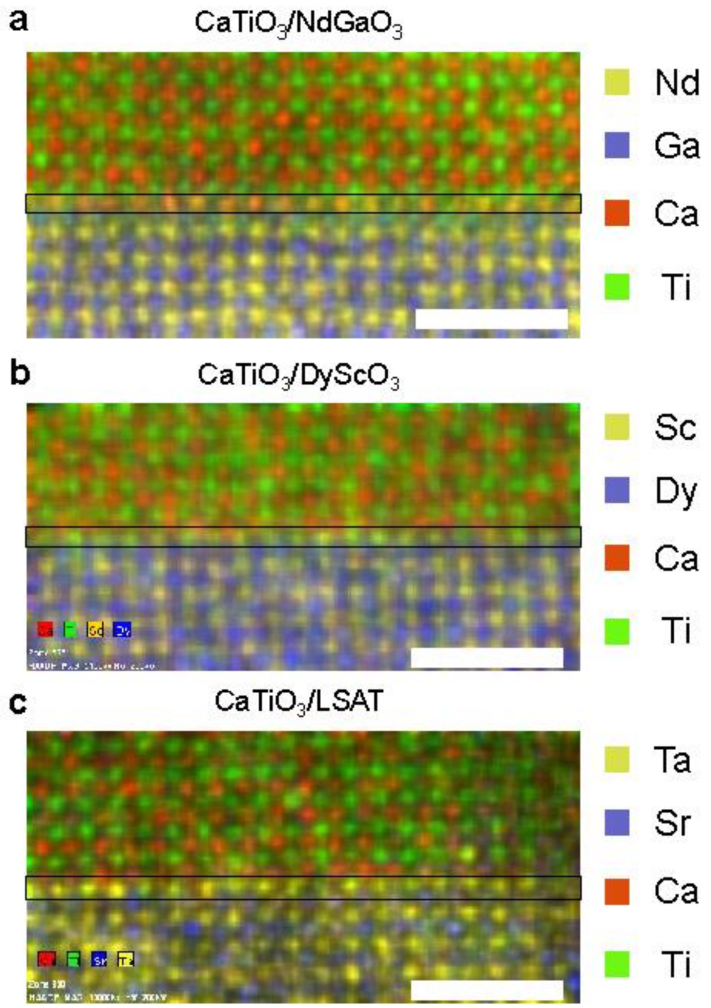

Supplementary Figure 5 | Energy dispersion spectrum mapping on the three systems. Energy dispersion spectrum (EDS) mapping on the three systems reveal termination of **a** NdO plane for $CaTiO_3/NdGaO_3$ **b** ScO_2 plane for $CaTiO_3/DyScO_3$, and **c** $Al_{0.65}Ta_{0.35}O_2$ plane for $CaTiO_3$/LSAT. The solid black boxes mark the surface layer of substrates. The scale bars are 2 nm.

Supplementary Table 2 | Octahedral tilt angles for different substrates and $CaTiO_3$ thin films averaged over the entire film. (RT: room temperature)

		α/º	β/º	γ/º	Tilt mismatch $\|\Delta\alpha\| + \|\Delta\beta\|$/º
Free bulk, Density functional theory (DFT)	$CaTiO_3$ (CTO)	9.09	9.18	9.09	-
	$NdGaO_3$ (NGO)	10.31	9.8	10.32	-
	$DyScO_3$ (DSO)	15	13	15	-
	LSAT	0	0	0	-
Strained $CaTiO_3$ bulk, DFT	1.1% (NGO)	9.37	9.53	8.15	1.21
	3.3% (DSO)	8.32	11.18	7.43	8.5
	1.2% (LSAT)	9.27	9.61	8.07	18.88
Epitaxial $CaTiO_3$, DFT	CTO/NGO	9.86	9.98	8.26	0.63
	CTO/DSO	11.04	10.63	7.76	6.33
	CTO/LSAT	3.92	4.66	8.1	8.58
Epitaxial $CaTiO_3$, COBRA 30K	CTO/NGO	9.91±0.35	9.53±0.35	7.71±0.35	0.67±0.49
	CTO/DSO	12.18±1.06	12.08±0.71	8.15±1.06	3.74±1.28
	CTO/LSAT	6.34±0.71	6.34±0.71	8.15±1.06	12.68±1.00
Epitaxial $CaTiO_3$, COBRA RT	CTO/NGO	12.00±1.06	10.50±0.35	7.90±1.06	2.39±1.12
	CTO/DSO	11.00±1.06	10.00±0.71	8.10±0.71	7.00±1.28
	CTO/LSAT	5.50±0.71	5.50±0.71	8.10±1.06	11.00±1.00

Supplementary Note 4: Room temperature EDs by COBRA and polarization by STEM

Room temperature EDs reconstructed by COBRA for $CaTiO_3/NdGaO_3$, $CaTiO_3/DyScO_3$, $CaTiO_3$/LSAT are respectively plotted in Supplementary Figures 6a-c. Detailed octahedral tilt angles for above three systems are shown in Supplementary Figures 6d, e and f. Due to the interfacial tilt epitaxy effect, α and β values show more gradual change across the interface compared to γ angles. Polarization components, P_a, P_b, P_c, in Supplementary Figures 6g and h reveal a small out-of-plane polarization, P_c (yellow dots), near the interfaces, due to the valence mismatch effect. The in-plane polarization components are zero for $CaTiO_3/NdGaO_3$, indicating its paraelectric state at room temperature. $CaTiO_3/DyScO_3$ has a small but non-zero polarization along *a*-axis (green dots in Supplementary Figure 6h), which indicates the system is close to its Curie temperature and consistent with SHG results shown in Fig. 5b. The valence mismatch effect is less clear in $CaTiO_3$/LSAT (yellow dots in Supplementary Figure 6i) due to the smallest valence mismatch value (-0.3) among all three systems as discussed in the main text. However, a clear in-plane polarization (green dots in Supplementary Figure 6i) with similar magnitude to 30 K results (Fig. 2f), reveals the $CaTiO_3$ on LSAT has the most stable ferroelectric state among all three systems. The Curie temperature of this system is much higher than room temperature (>900 K), as revealed by SHG measurement in Fig. 5b.

The STEM measurement was performed at room temperature to probe the polarization in these films. At room temperature, only the CTO film on LSAT displays significant polarization, which is demonstrated by the COBRA results in Supplementary Figure 6 and temperature dependent SHG results in Fig. 5b. However, we found that the polarizations extracted from STEM at room temperature were quite noisy. As shown in Supplementary Figure 7, the error bars of the polarization extracted from STEM on CTO/LSAT are quite significant when compared to the

mean values (solid lines). However, these mean values from STEM agree well with room temperature COBRA results (dots), suggesting non-zero polarizations in CTO along in-plane and out-of-plane directions. The fact that the COBRA error bar is smaller than that of STEM is probably due to the fact that X-ray diffraction employed in COBRA is macroscale in nature and the averaged structure information is being extracted over a large sample area.

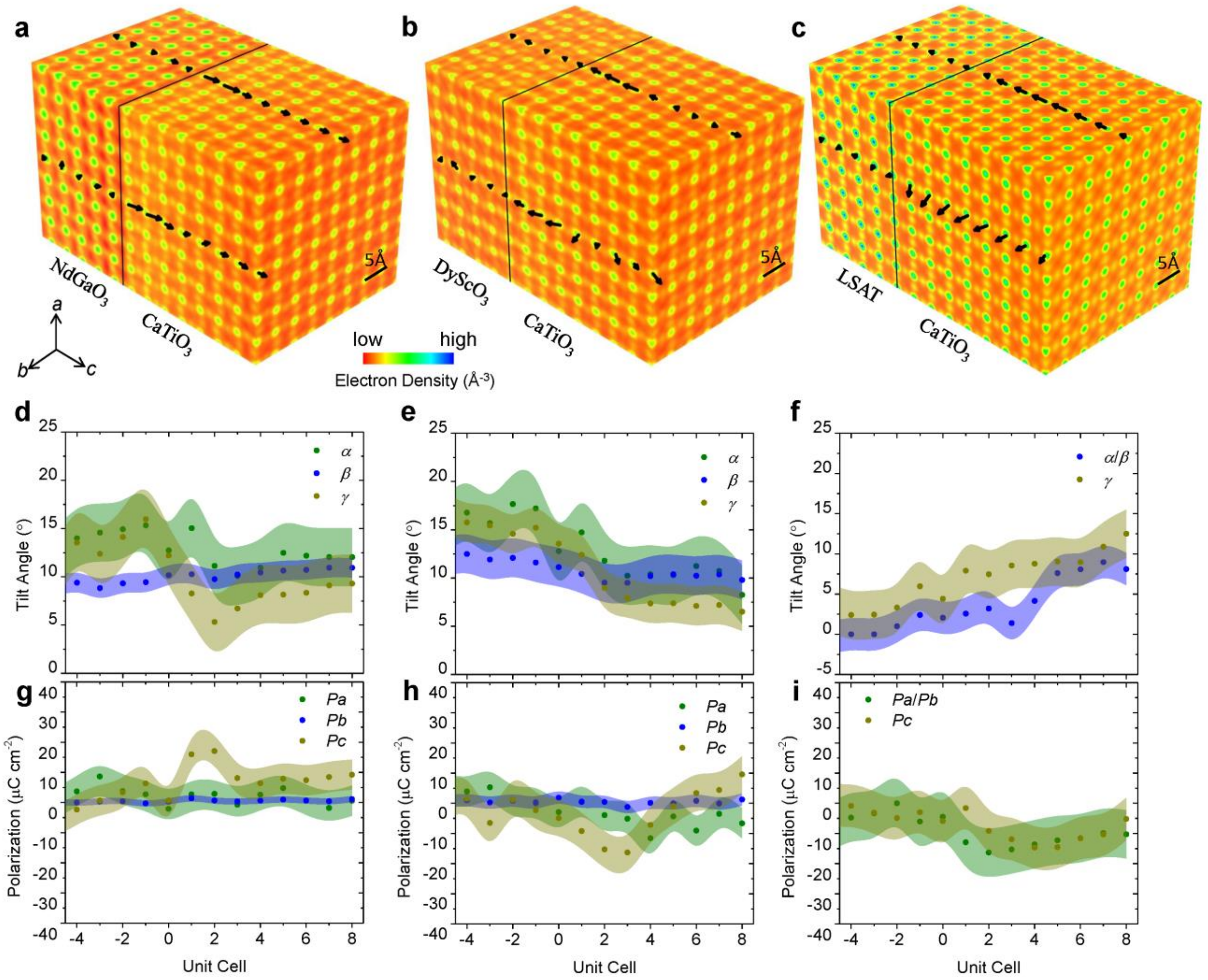

Supplementary Figure 6 | Three-dimensional electron densities reconstructed by coherent Bragg rods analysis at room temperature. Three-dimensional (3D) electron densities (EDs) for **a**

$CaTiO_3/NdGaO_3$ **b** $CaTiO_3/DyScO_3$ **c** $CaTIO_3$/LSAT at room temperature. One of the four equivalent $CaTiO_3$ domains on LSAT are plotted for convenience of comparison. In **d**, **e**, and **f**, quantified octahedral tilt angles, α (green), β (blue), γ (yellow), are shown, respectively. **g**, **h**, **i**, Polarization components, P_a (green), P_b (blue), P_c (yellow) extracted from COBRA (dots) are compared. The experimental errors are estimated by comparing substrates values to their reference bulk values and are indicated by the shaded area around the COBRA data dots.

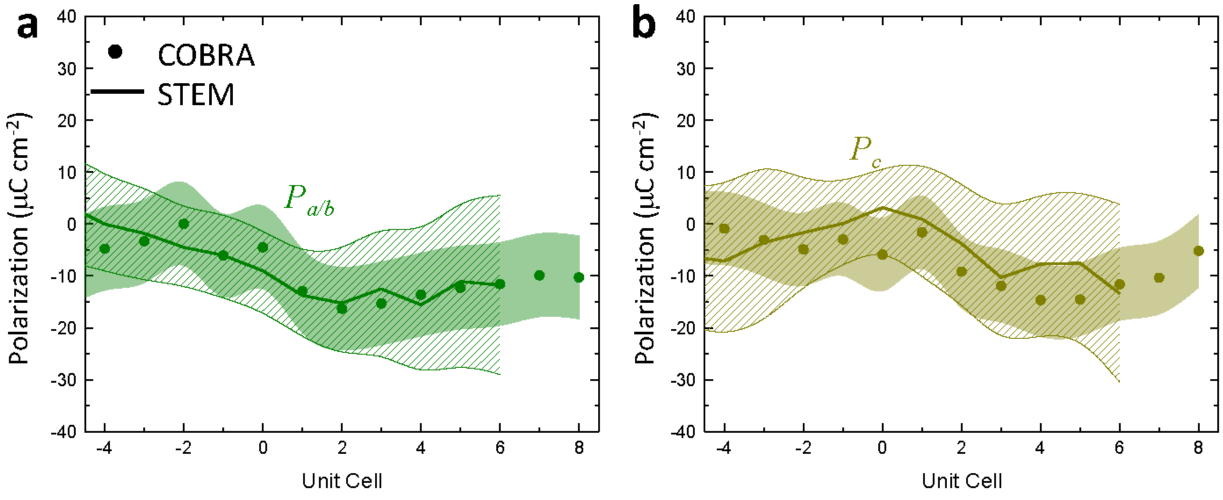

Supplementary Figure 7 | Room temperature polarization in $CaTiO_3$/LSAT. **a** In-plane and **b** out-of-plane polarization in $CaTiO_3$/LSAT at room temperature probed by scanning transmission electron microscopy (STEM) (solid lines) and coherent Bragg rods analysis (COBRA) (dots). The STEM error bars are taken to be the standard deviations over around 20 unit cells along the *a/b*-direction and are shown by the line shaded areas. The COBRA errors are estimated by comparing the substrate results to its bulk reference values, as shown by the color shaded areas.

Supplementary Note 5: [100] zone axis STEM images on $CaTiO_3$ on $NdGaO_3$ and $DyScO_3$

$CaTiO_3$, $NdGaO_3$ and $DyScO_3$ have out-of-phase tilts along [100] zone axis (a^- using Glazer notation). STEM images along [100] zone axis reveal two oxygen columns close to each other on oxygen sites, as illustrated by the left panel in Fig. 1a. The two oxygen columns in each pair are too close to each other that they show up as one broadened oxygen peak in STEM projected images, as shown in Supplementary Figure 8. By fitting the broadening of the oxygen peaks, the α angles can be extracted from these images and are plotted in green lines in Supplementary Figures 8c and d for $CaTiO_3$/$NdGaO_3$ and $CaTIO_3$/$DyScO_3$ respectively. However, the substrates values are much smaller than expected values of ~10.3° for $NdGaO_3$ and ~15° for $DyScO_3$. In contrast, room temperature COBRA reconstructed EDs on these two systems provides a 3D view of the oxygen octahedral tilts and overcomes the overlapping problem of the oxygen peaks. The α angles from COBRA method are plotted using green dots, which are much closer to the expected values for bulk substrates.

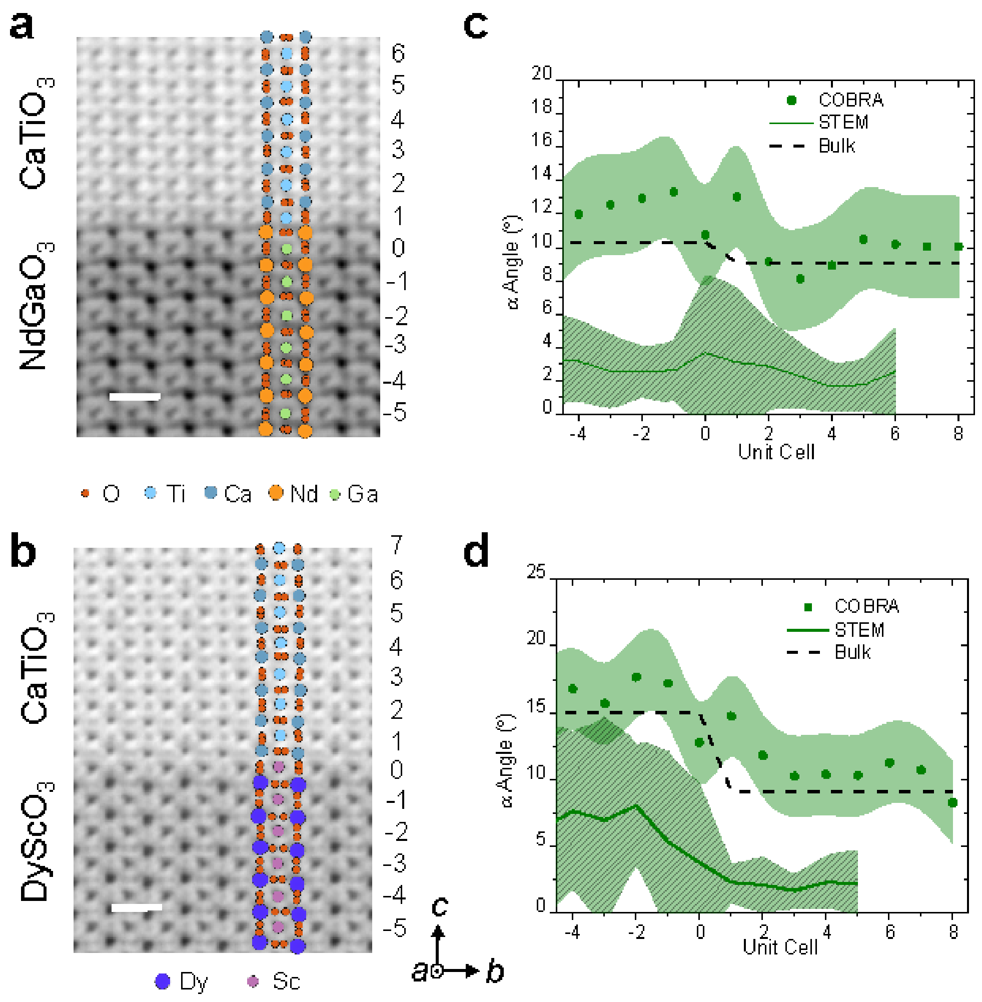

Supplementary Figure 8 | Room temperature STEM along [100] zone axis and α tilt angles. [100] zone axis scanning transmission electron microscopy (STEM) images for **a** $CaTiO_3/NdGaO_3$ and **b** $CaTiO_3/DyScO_3$ at room temperature reveal broadened oxygen peaks due to the out-of-phase tilt along *a*-axis. Different unit cells are numbered on the right side of the images. The scale bars are 5 Å. In **c** and **d**, α angles obtained by fitting the broadening of the oxygen peaks in STEM images (green lines) show smaller values than the expected bulk values (dashed lines). Room temperature coherent Bragg rods analysis (COBRA) (green dots) gives values much closer to that of bulk substrates, which are ~10.3° for $NdGaO_3$ and ~15.0° for $DyScO_3$. The STEM error bars are taken to be the standard deviations over around 20 unit cells

along the *b*-direction and are shown by the line shaded areas. The COBRA errors are estimated by comparing the substrate results to its bulk reference values, as shown by the green shaded areas.

Supplementary Note 6: [110] zone axis STEM images on $CaTiO_3$ on $NdGaO_3$, $DyScO_3$ and LSAT

Oxygen tilts can be probed by comparing the [110] zone axis STEM images with theoretical simulations, as proposed by Q.He, *et al*.[2] Samples of $CaTiO_3$/$NdGaO_3$, $CaTiO_3$/$DyScO_3$ and $CaTiO_3$/LSAT were thinning down to ~50 nm for STEM imaging. The detector collecting angles for the three systems were 9-50 mrad ($CaTiO_3$/$NdGaO_3$), 0–15 mrad ($CaTiO_3$/$DyScO_3$) and 1-15 mrad ($CaTiO_3$/LSAT). The experimental STEM images are shown in Supplementary Figure 9. Theoretical simulations (inserts) for $CaTiO_3$ used $a^-b^+c^-$ tilt pattern with tilt angles resolved by COBRA method, as listed in Supplementary Table 2. The experimental STEM images and simulations matches qualitatively well with each other, supporting the results of COBRA measurement. However, quantitative STEM analysis on the tilt angles requires resolving the subtle shapes of oxygen projection along [110] zone axis, which is still quite challenging.

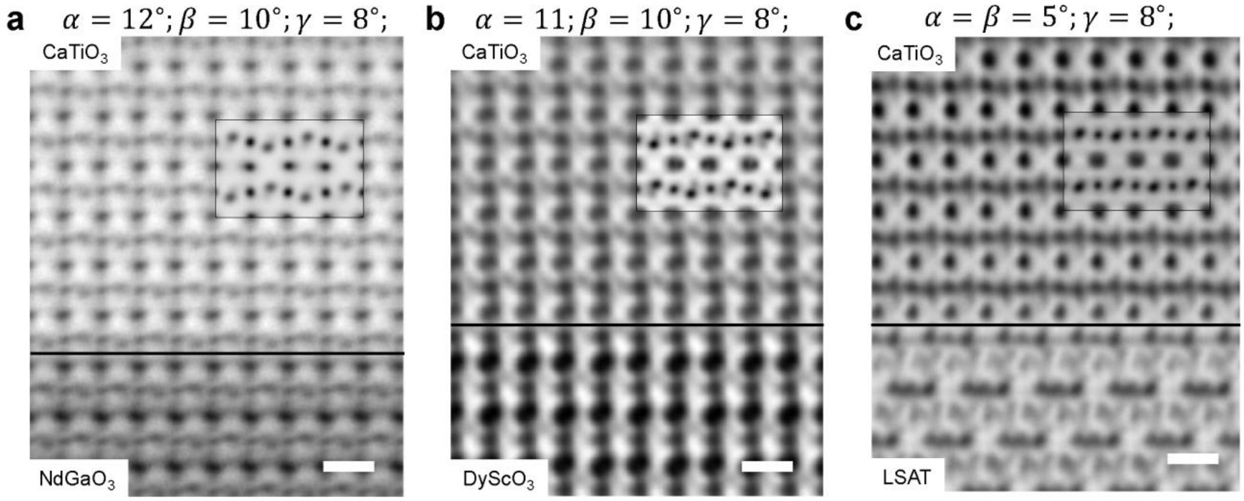

Supplementary Figure 9 | Room temperature STEM along [110] zone axis. [110] zone axis scanning transmission electron microscopy (STEM) images on **a** $CaTiO_3$/$NdGaO_3$, **b**

$CaTiO_3$/$DyScO_3$ and **c** $CaTiO_3$/LSAT at room temperature reveal broadened oxygen peaks due to the tilt of the oxygen octahedra. The theoretical simulated images (inserts) for $CaTiO_3$ with $a^-b^+c^-$ tilt pattern and COBRA resolved angles, which are specified above their images, are compared to the experimental images. The simulation and experiment qualitatively match with each other. The scale bars are 5 Å.

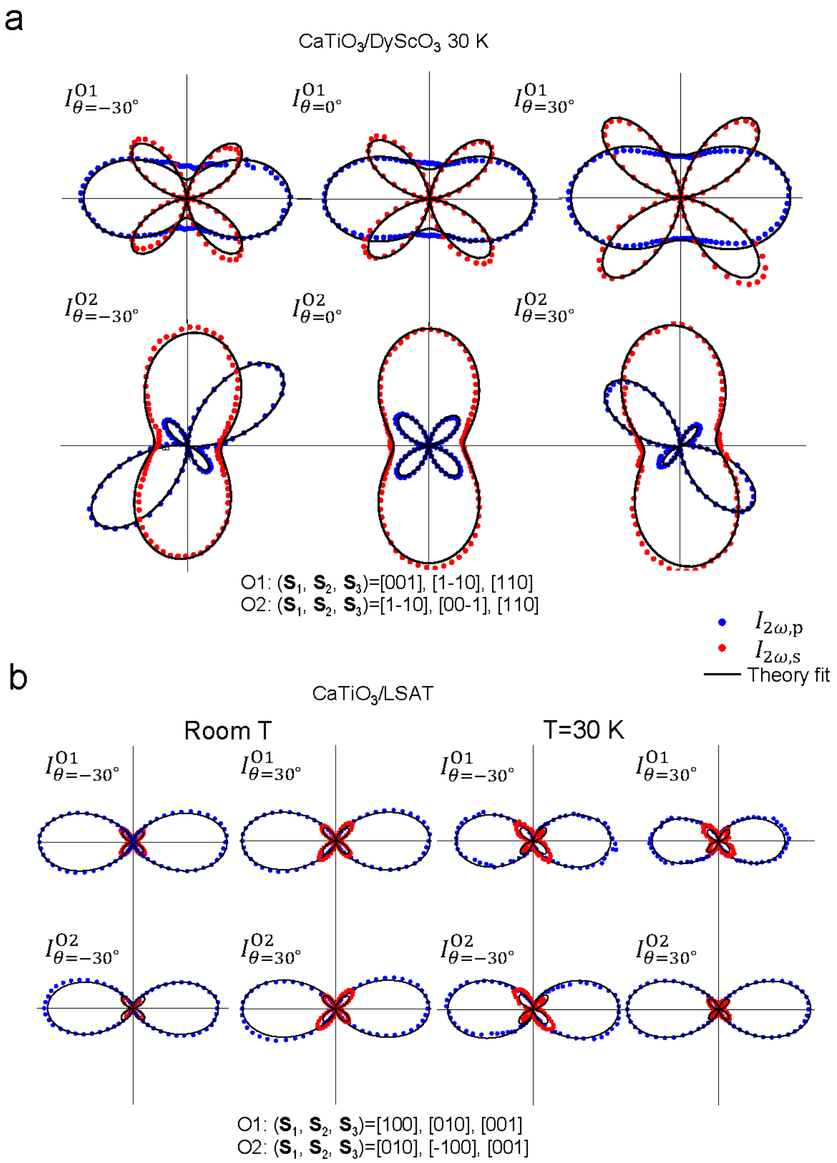

Supplementary Figure 10 | SHG polarimetry of $CaTiO_3$/$DyScO_3$ and $CaTiO_3$/LSAT.

Polarimetry data of p- (blue dots) and s- (red dots) polarized signal were measured under three incident angles θ=-30°, 0° and 30° and two sample orientations, O1 and O2, for **a** $CaTiO_3$/$DyScO_3$ at 30 K and **b** $CaTiO_3$/LSAT at room temperature and 30 K. Theoretical fitting (black lines) reveals a single domain monoclinic *m* symmetry for $CaTiO_3$/$DyScO_3$ at 30 K and four equivalent *m* domains for $CaTiO_3$/LSAT.

Supplementary Note 7: Termination of the $CaTiO_3$ films

The $CaTiO_3$ termination of the three systems can be seen clearly in the (110) pseudocubic planes as shown in Supplementary Figure 11, where alternating AO and BO_2 layers are directly visible. We can see that the CTO terminations are indeed same as their substrate terminations, giving an 8 u.c. of CTO films. We also notice that there is a weak electron density distribution above the outermost CTO layers. This suggests that there are incomplete atomic layers arising from the small imperfections during the growth control.

We also performed annular bright field (ABF) STEM on (110) pseudocubic zone axis on the three systems. However, we notice the topmost 1-2 u.c. of CTO films can be easily amorphized by the gold deposition on top of the film during the sample preparation process, which is intended for eliminating the charging effect of the electron beam. Thus, the termination of the CTO films cannot be revealed by STEM, as shown in Supplementary Figure 12.

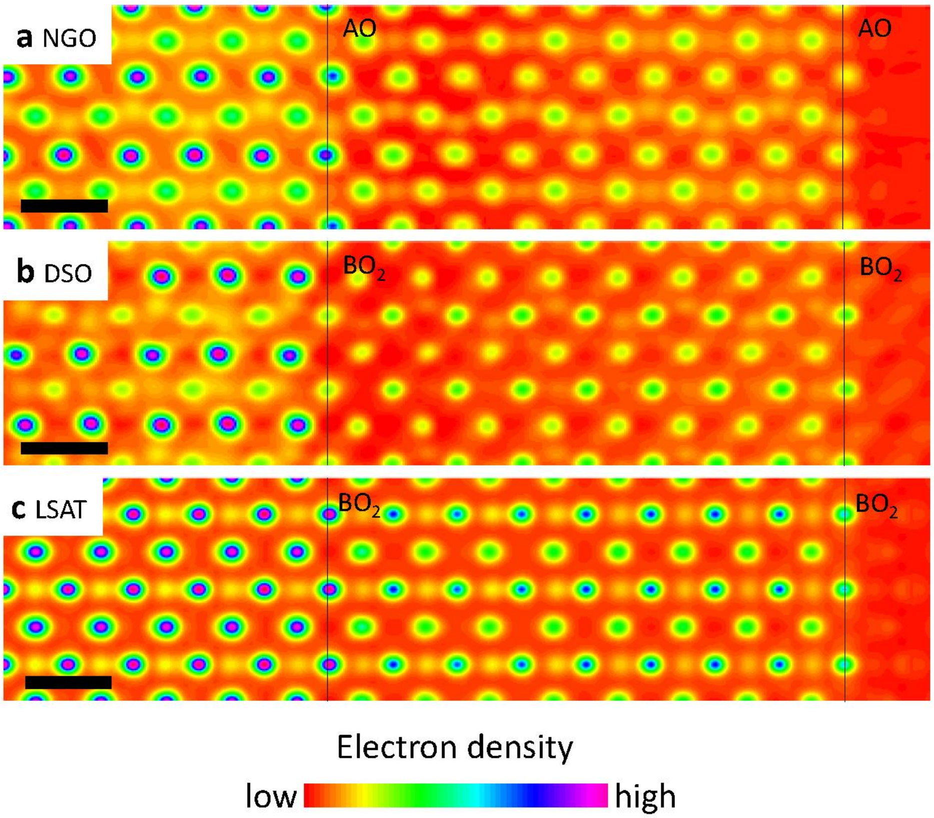

Supplementary Figure 11 | (110) slices of electron densities for three systems. (110) pseudocubic planes of coherent Bragg rods analysis (COBRA) reconstructed electron densities for **a** CTO/NGO **b** CTO/DSO and **c** CTO/DSO. Alternating AO and BO_2 layers can be clearly seen in this slice. These $CaTiO_3$ films have same terminating layer as their substrate, which indicates film thickness of 8 u.c.. The scale bars are 5 Å.

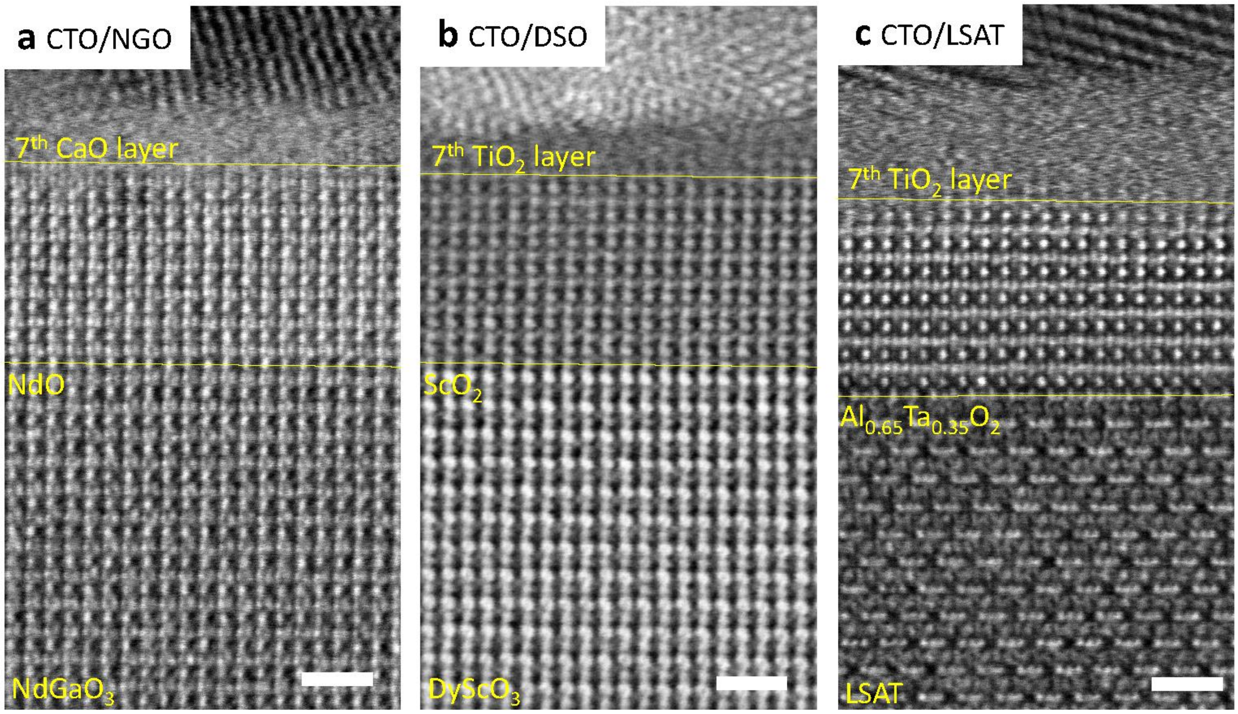

Supplementary Figure 12 | [110]-zone axis ABF-STEM images for three systems. [110] pseudocubic zone axis of annular bright field scanning transmission electron microscopy (ABF-STEM) images for **a** CTO/NGO **b** CTO/DSO and **c** CTO/DSO. The top most 1-2 u.c. of CTO layers are amorphized during the gold deposition process. The scale bars are 1 nm.

Supplementary Note 8: Discussion on initial model for COBRA iteration

In this note, we demonstrate that the final extracted angles are not sensitive to the initial model and the fits. The analysis process is detailed as following and demonstrated by the analysis on the data of CTO/NGO at 30 K.

First, we construct an initial model of CTO film with correct tilt pattern ($a^-b^+c^-$). This can be done by analyzing the half order peaks adopting the method discussed in Supplementary Note 3. As shown in Supplementary Figure 13, the combination of (1 2 5), (-1 -1 3), and (-1 3 3) peaks (under 2×2×2 pseudocubic notation) from $CaTiO_3$ clearly indicate a tilt pattern of $a^-b^+c^-$ for $CaTiO_3$ film on $NdGaO_3$ substrate.

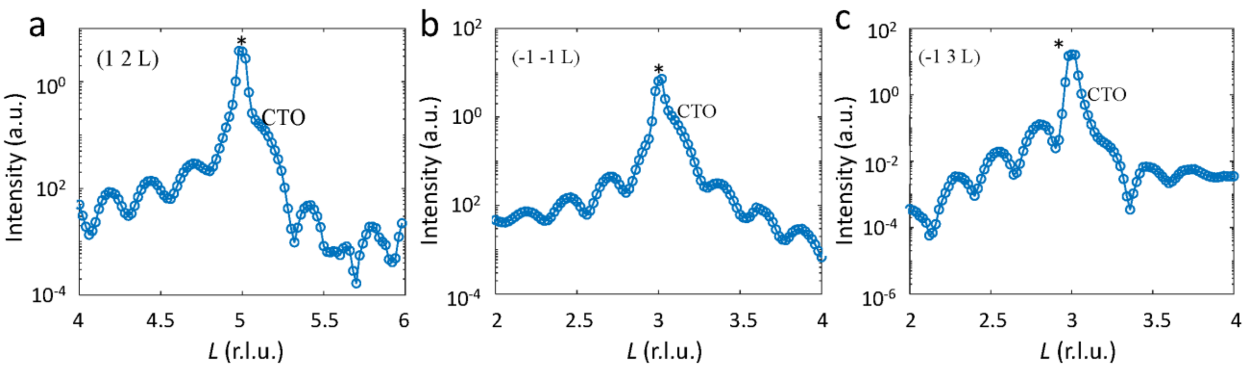

Supplementary Figure 13 | Half order peaks of CTO/NGO under 2×2×2 pseudocubic notation. a The presence of (1 2 5) peak from $CaTiO_3$ (CTO) film indicates an in-phase tilt about *b*-axis (b^+). The asterisk (*) marks the $NdGaO_3$ (NGO) substrate peak position. **b** The (-1 -1 3) peak of CTO film suggests either a^- tilt or b^- tilt. Since the tilt about *b*-axis has been determined to be b^+, the tilt about *a*-axis is out-of-phase (a^-). **c** Similarly, the (-1 3 3) peak from CTO suggests a c^- tilt about *c*-axis.

Then, we can choose different values for the three tilts angles of CTO. In this step, there is freedom in the choice of the angles. The analysis presented in the main text used bulk tilt angles of CTO as the starting point. Here, to demonstrate that the final results are not sensitive to the initial tilt angles, we used a different initial model with tilt angles of 7º, 5º, and 4º respectively for α, β, and γ. To overcome the stagnation during the COBRA iterations, we applied an atomicity constraint, which is to remove the unphysical features in the electron densities after every 10-20 iterations. The atomicity constraint was applied manually by constructing a new initial model for future iterations based on the atom positions yielded from previous iterations.[3] This process can be repeated multiple times until the results converge. Supplementary Figure 14 shows the tilts angles of all the intermediate states during the analysis on CTO/NGO. The COBRA analysis started with the initial model of 7º, 5º, and 4º respectively for α, β, and γ, producing an intermediate tilt state shown by Result 1 in Supplementary Figure 14. Then, we parameterized the atoms positions from the electron density of Result 1 and constructed a new starting model for the next 10-20 iterations, which produced Result 2, etc. After repeating this process for four times, the analysis converged to Result 4. As we can see in Supplementary Figure 14, the tilt angles yielded by these results are very close to the results presented in main Fig. 3a, which is labeled as Final in Supplementary Fig. 14.

These COBRA results can also be partially complemented by STEM study. As shown in main Fig. 4, the COBRA yielded β angles are consistent with the STEM results. This further supports the validity of the COBRA analysis.

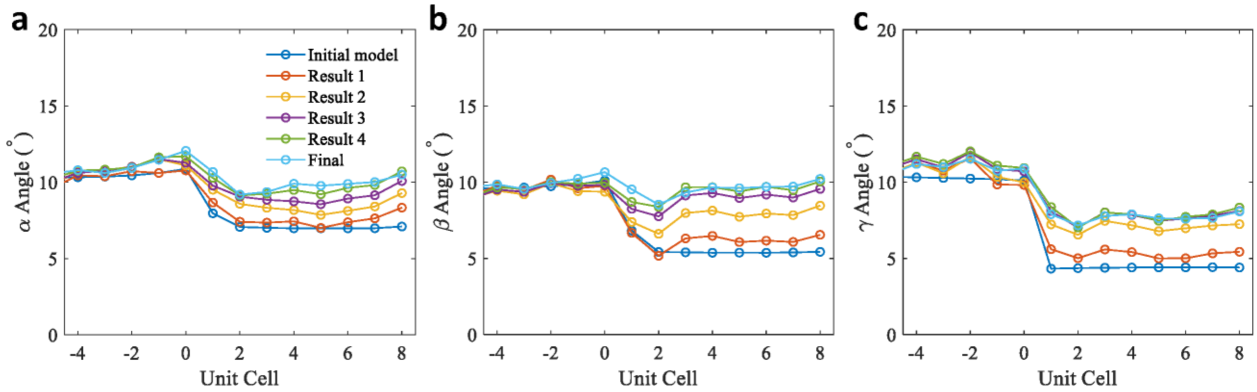

Supplementary Figure 14 | Test COBRA results with different initial model. a α, **b** β, and **c** γ values for all the intermediate states during the coherent Bragg rods analysis (COBRA) with initial model of 7º, 5º, and 4º respectively for α, β, and γ. The atomicity constraint was applied every 10-20 COBRA iterations to help the convergence of the results. The tilt angles given by Result 4 is considerably close to the results presented in the main text (labeled as Final), suggesting the COBRA results are robust towards different initial model.

Supplementary Note 9: Discussion on uncertainty analysis

To summarize this note, our method of error analysis (as detailed below) is more conservative that Zhou's method[4] (also described below).

We demonstrate this by comparing the uncertainties given by the above two methods for the case of CTO/NGO at 30 K. We first construct 8 sets of CTR data (Supplementary Figure 15) with simulated random noise adopting the method proposed by Zhou et al.[4], as shown in the plot below for (0 4) rod.

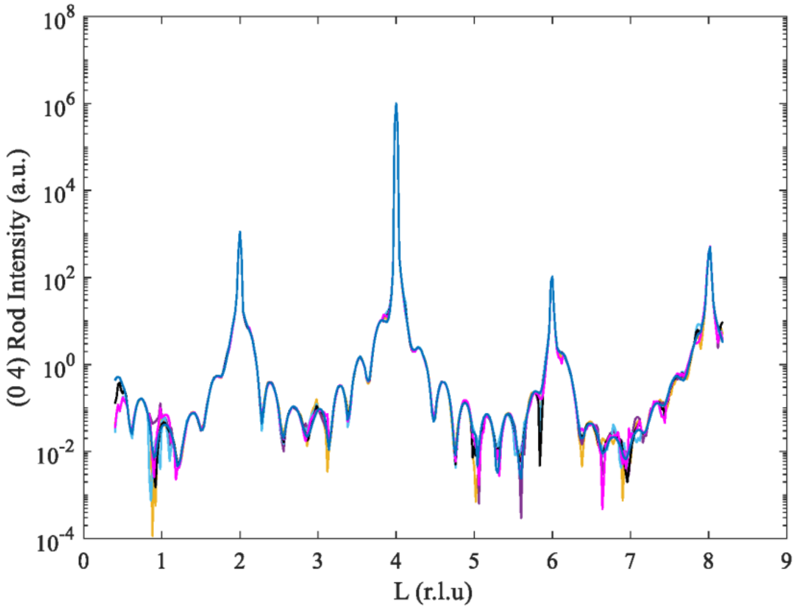

Supplementary Figure 15 | An example of crystal truncation rod with simulated noise. (0 4) crystal truncation rod of $CaTiO_3$/$NdGaO_3$ with 8 different sets of simulated noise are plotted as an example. The blue line is the original experimental measured (0 4) rod. Other colored lines are (0 4) rod with different set of simulated noise.

The COBRA results on these 8 sets of CTR data are plotted in the figure below (Supplementary Figure 16), where blue dots and their error bars are the results from the original experimental data and simplified uncertainties estimation proposed by this work. The red open circles are results based on 8 sets of CTR data with simulated noise. The grey area presents the statistical uncertainties of all the 8 data sets.

We can conclude that the uncertainties given by our simplified method provide an excellent conservative estimation of the upper limits of uncertainty in the COBRA results.

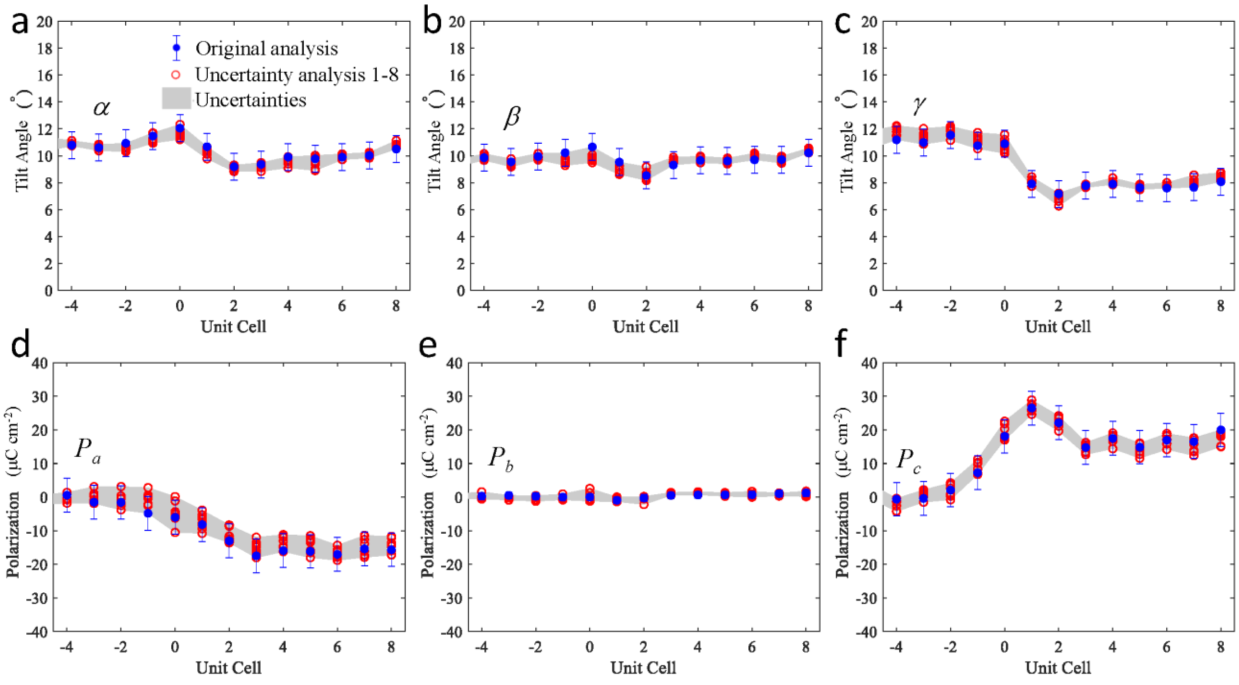

Supplementary Figure 16 | Comparison between two approaches of uncertainty analysis. Uncertainty analysis on $CaTiO_3$/$NdGaO_3$ at 30 K based on Zhou's bootstrap approach is plotted for tilt angles and polarizations using red open circles. The grey area represents the uncertainties determined from the scattering of the red open circles. The blue dots with error bars are original results using a simplified uncertainty estimation based on comparing the values for $NdGaO_3$ far

beneath the film-substrate interfaces with its bulk reference values. The blue error bars are close to the upper limit of uncertainties from bootstrap approach throughout the entire thickness.

More details on Zhou's bootstrap approach and our approach are discussed as follows. Since COBRA uses an iterative algorithm to reconstruct the real space electron density, traditional uncertainty analysis for fitting process, that based on a parameterized numerical model, is not applicable. Previously, Zhou et al, proposed a method based on the bootstrap approach to estimate the uncertainty of COBRA results. In their method, experimental CTR data are first randomly perturbed by simulated noise functions, and then analyzed by the COBRA method, yielding a new set of real space results. This process can be repeated for multiple times, which generates multiple sets of real space results. Finally, the uncertainties can be estimated by the scattering extent of these sets of results. This method is a systematic way of determining COBRA results' uncertainties, however, repeating COBRA iterations for a large number of times to gain statistics can be extremely time and effort consuming, especially for low symmetry systems of complex oxides, where a large unit cell and a large number of inequivalent atoms are involved. For example, in the cases of CTO/NGO and CTO/DSO presented by this work, positions of more than 350 atoms in 3D are needed for each analysis. Performing uncertainty analysis for all three systems at both room temperature and 30 K using the above bootstrap approach can be formidable.

Here we propose a straightforward way of estimating uncertainty as described in Method section. The deviation of atoms positions from bulk values in the first several unit cells in the substrate that are far beneath the substrate-film interfaces (> 5 u.c.) can be used as an estimation of uncertainties in COBRA analysis. Although this method gives an efficient way of estimating the

uncertainty, it might not reflect the fact that uncertainties may vary as going from substrate to film surface. Our analysis above indicates that this method is more conservative than Zhou's bootstrap method.

Supplementary References

1. Biegalski, M. D. *et al.* Impact of symmetry on the ferroelectric properties of $CaTiO_3$ thin films. *Appl. Phys. Lett.* **106,** 162904 (2015).

2. He, Q. *et al.* Towards 3D Mapping of BO_6 Octahedron Rotations at Perovskite Heterointerfaces, Unit Cell by Unit Cell. *ACS Nano* **9,** 8412–8419 (2015).

3. Fong, D. D. *et al.* Direct structural determination in ultrathin ferroelectric films by analysis of synchrotron x-ray scattering measurements. *Phys. Rev. B - Condens. Matter Mater. Phys.* **71,** 1–11 (2005).

4. Zhou, H., Pindak, R., Clarke, R., Steinberg, D. M. & Yacoby, Y. The limits of ultrahigh-resolution x-ray mapping: estimating uncertainties in thin-film and interface structures determined by phase retrieval methods. *J. Phys. D. Appl. Phys.* **45,** 195302 (2012).
